# Preservation of filtered environmental DNA samples at ambient high temperatures

**DOI:** 10.1016/j.mex.2026.103940

**Published:** 2026-05-03

**Authors:** Nao Matsumura, Qianqian Wu, Riko Matsuo, Masayuki K. Sakata, Toshifumi Minamoto

**Affiliations:** a Graduate School of Human Development and Environment, Kobe University, Japan 3-11, Tsurukabuto, Nada, Kobe, Hyogo 657-8501, Japan; b Research Faculty of Agriculture, Kita 9, Nishi 9, Kita-ku, Sapporo, Hokkaido 060-8589, Japan

**Keywords:** Aqueous eDNA, Buffer-ATL, Metabarcoding, Tropical fieldwork, Species-specific detection

## Abstract

Environmental DNA (eDNA) analysis is widely applied for biological monitoring; however, its use in tropical environments remains limited due to rapid eDNA degradation under high temperatures. This study evaluated the feasibility of storing eDNA samples at high-temperatures using Buffer ATL, a lysis buffer included in a commonly used extraction kit. After water filtration, 1 mL of Buffer ATL was added directly to Sterivex cartridge filters, which were stored at 20 °C and 40 °C for up to one week. The effectiveness of this method was evaluated using a tank experiment and natural river water samples. Fish eDNA concentration changes were quantified by species-specific qPCR, and changes in detected species richness were assessed by eDNA metabarcoding using MiFish primers. Adding Buffer ATL prevented eDNA decline during high-temperature storage and maintained fish species detection even after one week at 40 °C. This method provides a practical solution for preserving filtered eDNA samples during tropical fieldwork and transport under high-temperature conditions where cold storage is unavailable.

## Highlights

•Allows transport of eDNA filters at high temperatures by adding Buffer ATL.
•Remains fully compatible with downstream DNA extraction procedures.
•Maintains fish eDNA concentration and species detection for up to one week at 40 °C.

## Graphical abstract

**Figure:**
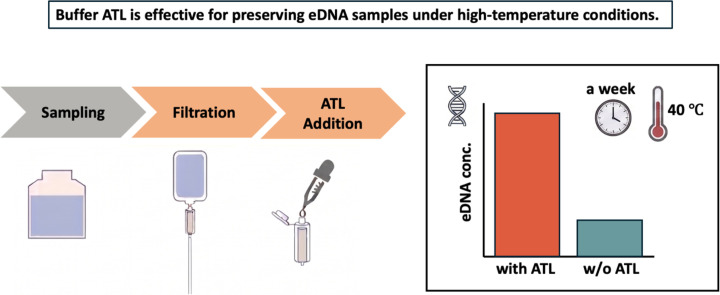

## Specifications table

**Table:** 

**Subject area**	Environmental Science
**More specific subject area**	Environmental DNA preservation and storage methods
**Name of your method**	ATL-based preservation of filtered environmental DNA samples under high-temperature conditions
**Resource availability**	Buffer ATL (Qiagen); Sterivex cartridge filters (0.45 µm)

## Background

The accelerating loss of biodiversity has become a global concern, highlighting the importance of continuous biological monitoring to track ecosystem changes [1]. Among various monitoring approaches, non-invasive methods are gaining attention. Environmental DNA (eDNA) analysis has emerged as a non-destructive and non-invasive technique for detecting organisms in aquatic and terrestrial environments. eDNA includes both intracellular and extracellular DNA derived from biological materials such as feces, mucus, gametes, and shed tissues [[2], [3], [4]]. By analyzing eDNA from water, soil, or air, researchers can assess species distribution, detect rare or invasive species, and monitor biodiversity [[4], [5]]. Among these applications, eDNA analysis using water as the sampling medium has become the most common and widely adopted approach.

While aqueous eDNA analysis has been widely applied in temperate rivers, oceans, and lakes (e.g. [[6], [7], [8]]), applications in tropical regions remain limited [9,10]. Tropical environments pose challenges for eDNA preservation due to high temperatures, which accelerate DNA degradation. Degradation is influenced by microbial activity and abiotic factors such as temperature, pH, and solar radiation [11,12].

To mitigate degradation, preservatives like cationic surfactants (e.g., benzalkonium chloride [BAC]) and buffers such as Longmire buffer have been used [13,14]. Additionally, lysis buffers included in DNA extraction kits may offer a practical solution for field preservation, though their effectiveness under tropical conditions remains unclear.

This study evaluates the preservation potential of Buffer ATL, a lysis buffer from the QIAGEN DNeasy Blood & Tissue Kit, under high-temperature conditions. Previous work has demonstrated that Buffer ATL is effective for preserving eDNA samples at ambient temperatures [15]. Using tank and river water samples, eDNA concentrations over time at 20 °C and 40 °C with and without ATL were compared using quantitative PCR (qPCR). For river samples, eDNA metabarcoding was also conducted to assess changes in fish species detection. These findings support the development of robust eDNA protocols for tropical fieldwork and long-distance sample transport under challenging conditions.

## Method details

Materials required for sampling and extraction of eDNA in this protocol are listed in Table 1.

**Table 1:** List of items required for this method.

Experimental step	ID	Name of the product	Supplier	Purpose
Water collection	1	Spout pouch (1000 ml)	COWPACK	Water collection, transportation, and gravity filtration
	2	10 % Benzalkonium chloride solution	Various manufacturers	Suppressing eDNA degradation
filtration	3	Cream double cap W-16	Kenis	Sealing the water sampling pack during filtration
	4	Repeat tie 150 mm	Various manufacturers	Securing the cream double cap
	5	Connecting tube	Nipro	Connecting to the Luer fitting
	6	Luer fitting VRM306	AS ONE	Connecting Sterivex cartridge (inlet) and the connecting tube
	7	Sterivex cartridge (0.45 µm)	Merck	Filtration
	8	Luer fitting VPRM406	AS ONE	Connecting Sterivex cartridge (outlet) and hose
	9	Luer fitting VRF306	AS ONE	Connecting Sterivec cartridge (outlet) and hose
	10	Vinyl Tube Hose 3 ×5	Various manufacturers	Connecting to the luer fitting VRF306
	11	Cable ties / S-hooks	Various manufacturers	Hanging the spout pouch
preservation	12	Luer fitting VRSP6	AS ONE	Capping the Sterivex cartridge (outlet)
	13	Dropper	Various manufacturers	Injecting ATL
	14	Buffer ATL	Qiagen	Preservation of eDNA
	15	Luer fitting VRMP6	AS ONE	Capping the Sterivex cartridge (inlet)

### Sampling and filtration of water and eDNA preservation using Buffer ATL

To prevent contamination, disposable gloves were worn during all experimental procedures. All instruments that directly contacted the samples were either brand-new or thoroughly cleaned using a commercially available chlorine-based bleach diluted to an effective chlorine concentration of approximately 0.1 %.

1. Water Sampling

A 500 mL water sample was collected from rivers or coastal areas and transferred into a spouted plastic pouch (1). The volume of collected water can be adjusted depending on environmental conditions and seasonal variations. To suppress eDNA degradation, 1/1000 vol of a 10 % BAC solution (2) was added to the water sample, resulting in a final concentration of 0.01 % [14].

2. Filtration

Following Oka et al [16], gravity filtration was performed using a Sterivex cartridge filter (0.45 µm). The spout of the pouch (1) was sealed with a cream double cap (3) and secured with a repeat tie (4). The following components were connected in sequence using luer fittings: Nipro connector tube (5), VRM306 (6), Sterivex cartridge (7), VPRM406 (8), VRF306 (9), and a 1.5 m vinyl tube hose (10). During filtration, the pouch was suspended approximately 2 m above ground using cable ties and an S-hook (11), then inverted and left until the water had completely drained. Filtration typically took several minutes to several hours.

3. Buffer Addition

After confirming that no water remained inside the Sterivex cartridge, the outlet was sealed with a luer fitting VRSP6 (12). Using a pipette (13), 1 mL of Buffer ATL (14) was injected into the Sterivex cartridge, which was then gently rotated to ensure thorough distribution of the buffer. Finally, the inlet was sealed with another VRSP6 fitting (15).

4. Storage Conditions

Under these conditions, eDNA can be stably recovered even after storage at temperatures up to 40 °C for one week.

## Method validation

Sampling was conducted for method validation rather than for assessing ecological representativeness. Although seasonal variation was not explicitly examined, the preservation performance of Buffer ATL is based on its intrinsic physicochemical properties, and the method is expected to be applicable across seasons when similar temperature conditions are encountered.

Validation 1 used environmental water from a tank housing *Hemigrammocypris neglecta*, and Validation 2 used water from the Sumiyoshi River, where *Plecoglossus altivelis* are present. For each sample, water collection, filtration, post-filtration storage, and DNA extraction were performed as above. Subsequently, qPCR was used to compare changes in eDNA concentration over time between filters with and without ATL addition. For post-filtration storage, samples were kept for different durations at 20 °C or 40 °C. Furthermore, environmental water samples were analyzed by eDNA metabarcoding using MiFish primers [17] to assess changes in detected fish species over time depending on ATL addition.

Validation 1: Tank Experiment

### Tank setup and DNA extraction

Four tanks were prepared with 20 L of dechlorinated water four days prior to sampling. Three tanks each contained five adult *H. neglect* (3–5 cm in standard length), and one tank served as a field blank.

From each tank, 12 L of water was collected, and 12 mL of 10 % BAC solution was added to prevent DNA degradation, followed by thorough mixing [14]. Then ten replicate filtrations were performed for each tank. For a single filtration, 500 mL of tank water was filtered using Sterivex cartridges by gravity filtration following Oka et al [16]. A plastic bag was placed approximately 2 m above ground, and filtration continued until water completely drained (within 24 h). After filtration, Sterivex cartridges were divided into two groups: with 1 mL ATL added and without ATL. Samples were then stored under five conditions: immediately frozen (0 h), 20 °C for 10 h, 20 °C for 1 week, 40 °C for 10 h, and 40 °C for 1 week. After the storage period, all samples were kept at –25 °C until extraction.

DNA extraction from Sterivex cartridges followed the Buffer ATL-based method described by Wu and Minamoto [15] using the DNeasy Blood & Tissue Kit (Qiagen). For Sterivex cartridges without ATL, 1 mL ATL was added at this stage. Then, 1 mL of lysis mixture (495 µL 1 × PBS, 455 µL Buffer AL, 50 µL Proteinase K) was added to each cartridge and incubated at 56 °C for 30 min. After incubation, cartridges were centrifuged at 6000 × *g* for 3 min, and 1 mL ethanol was added to the eluate and mixed thoroughly. The solution was transferred to DNeasy columns in ∼650 µL aliquots and centrifuged at 6000 × *g* for 1 min. Flow-through was discarded, and the column was reloaded with remaining solution until all liquid passed through. DNA was then purified following the DNeasy Blood & Tissue Kit protocol and eluted in 150 µL Buffer AE.

### Quantitative PCR

Quantitative PCR was performed using a QuantStudio 3 Real-Time PCR System (Thermo Fisher Scientific). For the tank experiment, *H. neglect* was targeted, and primers and probe were designed for the Cytb region within the mitochondrial genome (Table 2; [18]).

**Table 2:** Primers and probes used in this study.

Assay	Name of primers / probes	Sequence (5´ > 3´)
qPCR (*Hemigrammocypris neglect*)	Hra-cytB-F	CACCCCAGCAAACCCCTTA
	Hra-cytB-R	ACTAGAATAGAGAACAGTAACGCGAGAA
	Hra-cytB-P	FAM- TGTTCGCTTACGCCATT -MGB-NFQ
qPCR (*Plecoglossus altivelis*)	Paa-cytB-F	CCTAGTCTCCCTGGCTTTATTCTCT
	Paa-cytB-R	GTAGAATGGCGTAGGCGAAAA
	Paa-cytB-P	FAM- ACTTCACGGCAGCCAACCCCC -TAMRA
MiFish metabarcoding (1st PCR)	MiFish-U-F	ACACTCTTTCCCTACACGACGCTCTTCCGATCTNNNNNNGTCGGTAAAACTCGTGCCAGC
	MiFish-U-R	GTGACTGGAGTTCAGACGTGTGCTCTTCCGATCTNNNNNNCATAGTGGGGTATCTAATCCCAGTTTG
MiFish metabarcoding (2nd PCR)	2nd Forward	AATGATACGGCGACCACCGAGATCTACAXXXXXXXXACACTCTTTCCCTACACGACGCTCTTCCGATCT
	2nd Reverse	CAAGCAGAAGACGGCATACGAGATXXXXXXXXGTGACTGGAGTTCAGACGTGTGCTCTTCCGATCT

N represents random nucleotides. Eight consecutive 'X' characters represent a sample-specific index.

Each PCR reaction (20 µL total volume) contained 1 × TaqMan Environmental Master Mix 2.0 (Thermo Fisher Scientific), 0.1 µL AmpErase (Thermo Fisher Scientific), 2 µL DNA template, 900 nM forward and reverse primers, and 125 nM probe, with ultrapure water added to reach the final volume.

For each PCR run, quantitative standards consisting of synthetic DNA at 30, 300, 3000, and 30,000 copies per reaction were analyzed in triplicate. Non-template controls (ultrapure water instead of template DNA) were also included in triplicate.

Thermal cycling conditions were as follows: 50 °C for 2 min, 95 °C for 10 min, followed by 55 cycles of 95 °C for 15 s and 60 °C for 1 min. PCR was performed in triplicate, and eDNA concentrations were calculated as the mean of three replicates. If any replicate showed no amplification, its concentration was treated as zero when calculating the mean.

### Quantitative PCR results and effectiveness of ATL in tank samples

Environmental DNA was detected under all conditions, regardless of ATL addition. No amplification was observed in field blanks or PCR non-template controls. For eDNA concentrations detected in the tanks housing *H. neglect*, the values were 104,745 ± 43,587 (mean ± SD) copies/reaction with ATL and 86,387 ± 52,478 copies/reaction without ATL (Appendix Table 1).

**Fig. 1:**
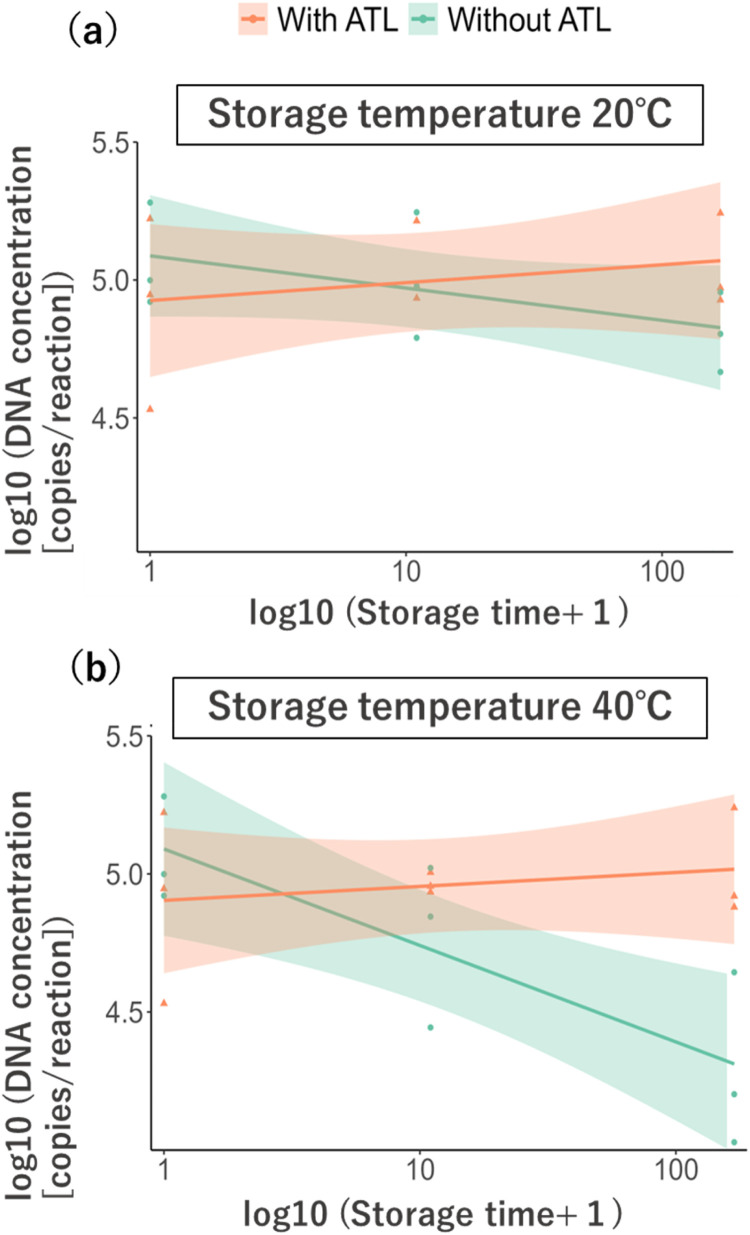
Results of environmental DNA yield with and without ATL addition (Validation 1: Tank experiment). The temporal changes in environmental DNA concentration at a storage temperature of (a) 20 °C and (b) 40 °C. The confidence interval for the regression line is 95 %.

To compare changes in eDNA concentration over time between Sterivex cartridges with and without ATL under post-filtration storage at 20 °C and 40 °C, statistical analysis was performed using a linear mixed-effects model. The model was fitted with the *lmer* function in the R package *lme4*, where the response variable was the log-transformed eDNA concentration (copies/reaction) of *H. neglect*. Fixed effects included ATL addition (yes/no) and the interaction with log-transformed storage time (+1), while random effects included storage temperature and tank replicate. Data from 0 h were used in both 20 °C and 40 °C analyses. Results showed that when ATL was not added to Sterivex cartridges, eDNA yield significantly decreased over time (*p* < 0.001; Fig. 1, Table 3).

**Table 3:** Linear mixed model (LMM) results showing the effect of ATL addition on the preservation of filtered samples (tank experiment: eDNA concentration of *Hemigrammocypris neglect*).

	coefficient	Std error	*t*-value	*p*-value	significance
(Intercept)	4.9475	0.1371	36.0850	2.13E-05	⁎⁎⁎
preservation period	0.0005	0.0005	0.9830	0.3336	
ATL addition (vs. no ATL)	0.0454	0.0712	0.6380	0.5284	
Interaction	−0.0032	0.0007	−4.3300	0.0002	⁎⁎⁎

⁎⁎⁎ *p* ≤ 0.001.

Validation 2: River Water Experiment

### Sampling and DNA extraction

River water was collected near the estuary of the Sumiyoshi River, an approximately 8 km river flowing through eastern Kobe, Japan. On July 9, 2024, a total of 20 L was sampled using two 10 L polyethylene tanks. To prevent DNA degradation, 10 mL of 10 % BAC solution was added to each tank and mixed thoroughly. As a field blank, 500 mL of purified water was transported to the sampling site and treated with BAC in the same manner. Samples were then brought back to the laboratory and filtered by gravity using Sterivex cartridges (500 mL each), with nine replicate filters for each of the four treatments (with or without ATL at 20 °C or 40 °C). Subsequent steps from filtration to DNA extraction were identical to those described for the tank experiment.

### Quantitative PCR

For river water samples, qPCR targeted *P. altivelis*, a species known to inhabit the river, using primers and probe for the mitochondrial Cytb region (Table 2; [19]). Except for the primers and probe, all procedures were identical to those used for *H. neglect* in the tank experiment.

### Quantitative PCR results and effectiveness of ATL in river samples

Environmental DNA was detected under all conditions, regardless of ATL addition. No amplification was observed in field negative blanks or non-template controls. For eDNA concentrations of *P. altivelis* detected in samples from the Sumiyoshi River, the values were 919 ± 144 (mean ± SD) copies/reaction with ATL and 738 ± 248 copies/reaction without ATL (Appendix Table 2).

**Fig. 2:**
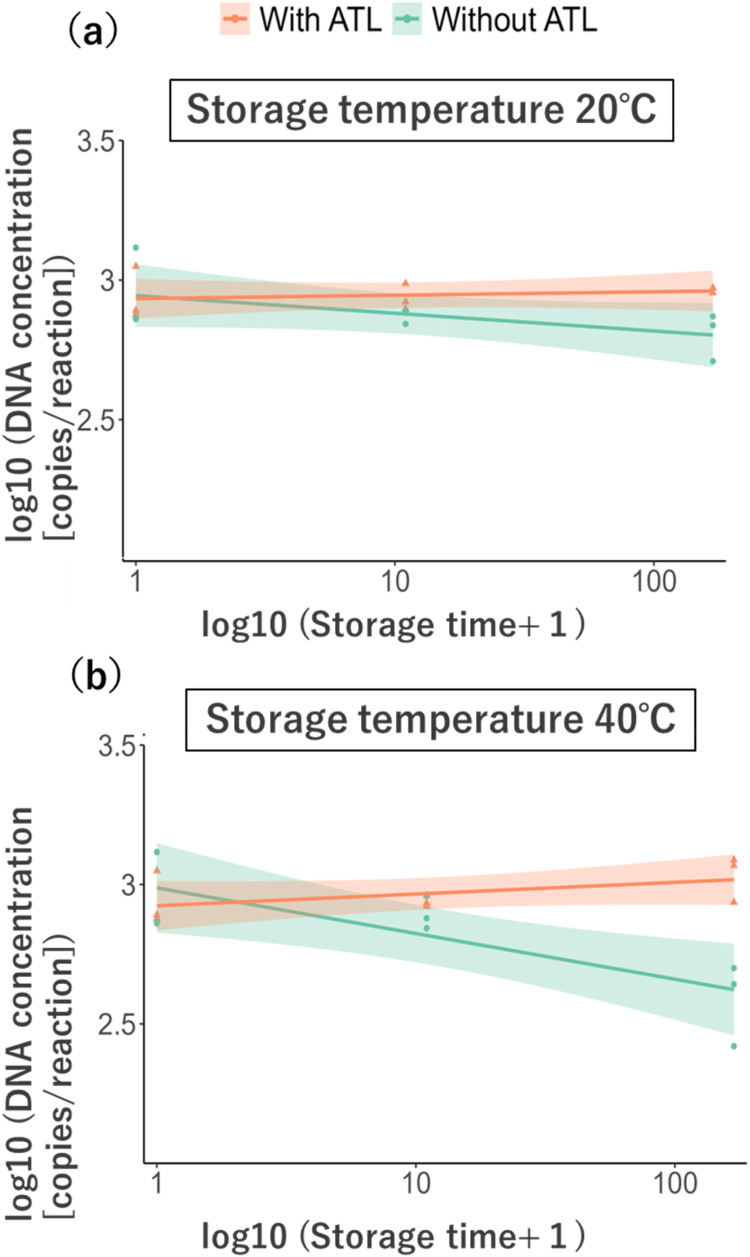
Results of environmental DNA yield with and without ATL addition (Validation 2: Field samples from the Sumiyoshi River). The temporal changes in environmental DNA concentration at a storage temperature of (a) 20 °C and (b) 40 °C. The confidence interval for the regression line is 95 %.

To compare changes in eDNA concentration over time between Sterivex cartridges with and without ATL under post-filtration storage at 20 °C and 40 °C, statistical analysis was performed using a linear mixed-effects model. The model was fitted with the *lmer* function in the R package *lme4*, where the response variable was the log-transformed eDNA concentration (copies/reaction) of *P. altivelis*. Fixed effects included ATL addition (yes/no) and its interaction with log-transformed storage time (+1), while random effects included storage temperature and sampling replicate. Results showed that when ATL was not added to Sterivex cartridges, eDNA yield significantly decreased over time (*p* < 0.01; Fig. 2, Table 4).

**Table 4:** Linear mixed model (LMM) results showing the effect of ATL addition on the preservation of filtered samples (river experiment: eDNA concentration of *Plecoglossus altivelis*).

	coefficient	Std error	*t*-value	*p*-value	significance
(Intercept)	2.9363	0.0309	95.0020	1.68E-13	⁎⁎⁎
preservation period	0.0004	0.0003	1.1540	0.2818	
ATL addition (vs. no ATL)	−0.0135	0.0437	−0.3090	0.7649	
Interaction	−0.0017	0.0004	−3.8310	0.0050	⁎⁎

⁎⁎⁎ *p* ≤ 0.001.

⁎⁎ *p* ≤ 0.01.

### Fish eDNA metabarcoding

To examine changes in the number of detected fish species between samples with and without ATL addition after filtration, eDNA metabarcoding using MiFish-U primers [17] was performed. DNA amplification and tag addition for sequencing were conducted using a two-step PCR approach. Library preparation followed the protocol described by Wu and Minamoto [15] with slight modification. The first PCR was performed in four replicates to reduce false negatives, using KOD Plus Neo (Toyobo, Japan) as the DNA polymerase. The PCR reaction mixture (12 µL total volume) consisted of 0.25 µL KOD Plus Neo, 1 × PCR Buffer, 250 nM dNTP mix, 1.56 mM MgSO₄, 625 nM of each primer, 2 µL of five-fold diluted DNA template, and ultrapure water. The thermal cycling conditions were as follows: initial denaturation at 95 °C for 30 s, followed by 40 cycles of denaturation at 98 °C for 10 s, annealing at 60 °C for 10 s, and extension at 72 °C for 30 s, and a final extension at 72 °C for 5 min. Individual first PCR products were pooled by sample and stored at –25 °C until the next step. For each PCR run, four non-template controls were included, and field blanks were processed through all steps up to next-generation sequencing. Sequencing was performed using the iSeq 100 System (Illumina).

A total of 1483,747 raw reads were obtained. Data processing and analysis were conducted using USEARCH v10.0.240 [20] following the method of Wu et al [21]. To remove potential contamination, the number of reads detected in field blanks and PCR blanks was subtracted from each corresponding sample. In addition, nine non-target mammal species, including *Homo sapiens* were excluded from subsequent analyses. After removing these non-target taxa, 1115,667 reads were retained for fish species analysis.

### Metabarcoding results and effectiveness of ATL in river samples

A total of 24 freshwater and marine fish species were detected (list of detected species: Appendix Table 3; number of species per sample: Appendix Table 4; read counts per sample: Appendix Tables 5 and 6).

**Fig. 3:**
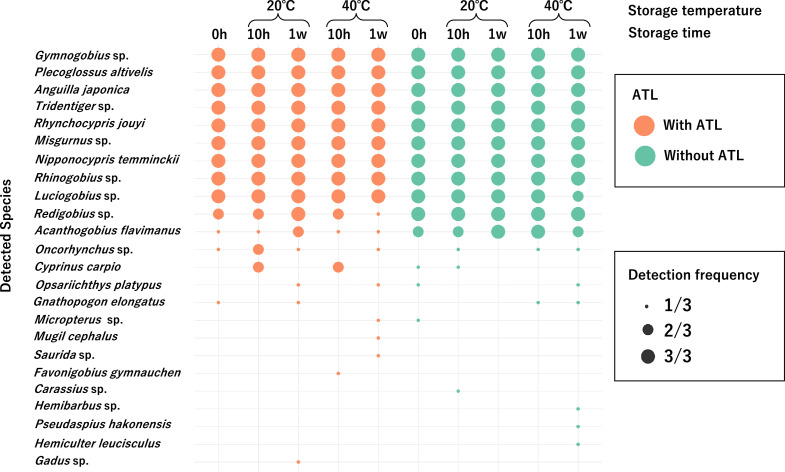
Results of the number of detected fish species with and without ATL addition (Validation 2: Field samples from the Sumiyoshi River).

To examine changes in the number of detected fish species over time between Sterivex cartridges with and without ATL under post-filtration storage at 20 °C and 40 °C, statistical analysis was performed using a generalized linear mixed-effects model with a Poisson error distribution. The model was fitted with the *glmer* function in the R package *lme4*, where the response variable was the number of fish species detected by MiFish metabarcoding. Fixed effects included ATL addition (yes/no) and its interaction with log-transformed storage time (+1), while random effects included storage temperature and sampling replicate. Results showed that the number of detected fish species did not change significantly over time depending on ATL addition (Fig. 3, Table 5).

**Table 5:** Generalized linear mixed model (GLMM) results showing the effect of ATL addition on the preservation of filtered samples (river experiment: number of detected species by metabarcoding).

	coefficient	Std error	*z*-value	*p*-value	significance
(Intercept)	2.3876	0.0901	26.4930	<2e-16	⁎⁎⁎
preservation period	0.0004	0.0009	0.4620	0.6440	
ATL addition (vs. no ATL)	0.0620	0.1256	0.4940	0.6210	
Interaction	−0.0004	0.0013	−0.2970	0.7660	

⁎⁎⁎ *p* ≤ 0.001.

### Summary of validation results

This study evaluated the effectiveness of using Buffer ATL for preserving eDNA samples under high-temperature conditions, such as those encountered in tropical regions, to prevent degradation and loss of DNA yield. Specifically, the impact of ATL addition on eDNA yield and the number of detected fish species over time were assessed.

Results from experiments measuring eDNA yield showed that when ATL was not added, DNA yield decreased significantly over time. In contrast, ATL addition maintained eDNA yield even after one week of storage at temperatures up to 40 °C, with no difference compared to samples immediately frozen at –25 °C. These findings were consistent for both tank and river water samples, demonstrating that ATL-based preservation is effective not only under controlled conditions but also in field environments. This suggests that ATL enables temporary storage of filtered samples at ambient temperature in tropical regions, even where stable power supply for freezing is unavailable or unstable.

To simulate field conditions, BAC was added to collected water prior to filtration, and the effect of ATL addition to Sterivex cartridges was tested. Previous research demonstrated that adding 0.01 % BAC to water suppresses eDNA degradation during storage at ambient temperature without on-site filtration [14]. In our study, however, filters obtained by filtering BAC-treated water and stored at 40 °C without ATL showed pronounced eDNA degradation (Figs. 1b, 2b). This indicates that BAC alone is insufficient for long-term storage under high temperatures, and ATL addition to filters can further prevent eDNA degradation during transport in tropical environments.

For metabarcoding analysis, river samples were used to examine changes in detected fish species over time with and without ATL. Results showed no difference in species richness between samples stored for one week at 40 °C with ATL and those immediately frozen (Fig. 3; Appendix Table 4). Interestingly, species richness did not decrease over time even without ATL. Wu and Minamoto [15] reported that although ATL increased eDNA yield compared with RNAlater, it did not lead to differences in species detection. These findings suggest that addition of BAC may have contributed to stable species detection regardless of ATL. However, given that DNA yield decreased without ATL, prolonged storage beyond one week [22] or detection of rare species may be compromised. Therefore, ATL-based preservation is recommended not only for quantitative assessments but also for metabarcoding applications.

Because Buffer ATL is the initial reagent used in Qiagen’s DNA extraction protocol, its use as a preservation solution allows seamless transition to downstream extraction without additional pretreatment steps.

## Limitations

In this study, the proposed method was validated only in freshwater environments. Therefore, its effectiveness in brackish and marine waters needs to be further examined. In addition, no validation was conducted using filters other than Sterivex cartridges. Membrane filters are also commonly used for eDNA filtration, and it would be beneficial to confirm the applicability of this method when such filters are employed.

## Ethics statements

This study involved tank experiments using fish. Under current Japanese law, there are no specific regulations or mandatory ethical review requirements for animal experimentation involving fish. Nevertheless, all experimental procedures in this study were conducted in accordance with the Animal Experimentation Guidelines of Kobe University, and the required institutional notification was properly submitted (No. 2023-05). In addition, all experiments complied to the ARRIVE (Animal Research: Reporting of In Vivo Experiments) guidelines.

## CRediT author statement

**Nao Matsumura**: Conceptualization, Methodology, Data acquisition, Experiment, Formal analysis, Writing – original draft, Writing –review & editing. **Qianqian Wu**: Conceptualization, Methodology, Data acquisition, Writing –review & editing. **Riko Matsuo**: Data acquisition, Experiment, Writing –review & editing. **Masayuki K. Sakata**: Formal analysis, Writing –review & editing. **Toshifumi Minamoto**: Supervision, Funding acquisition, Conceptualization, Writing – original draft, Writing –review & editing.

## Declaration of interests

The authors declare the following financial interests/personal relationships which may be considered as potential competing interests:

Toshifumi Minamoto is an inventor of the use of BAC for preserving environmental DNA in water samples.

## Data Availability

Data will be made available on request.

## Appendix Supplementary materials

Image, application 1
