# Supplementary material for: Preservation of filtered environmental DNA samples at ambient high temperatures
Source: MethodsX. 2026 May 3;16:103940. doi: 10.1016/j.mex.2026.103940 (PMC13196014; doi:10.1016/j.mex.2026.103940)
Supplement: Supplementary file 1 [file mmc1.docx]

Appendix Table 1. The results of quantitative PCR for tank experiments (Validation 1).

| ID | ATL | Preserved temperature  [℃] | Preserved time  [h] | # Tank | eDNA concentration  [copies/reaction] |
| --- | --- | --- | --- | --- | --- |
| 1 | + | 20 | 0 | 1 | 88,356 |
| 2 | + | 20 | 10 | 1 | 85,880 |
| 3 | + | 20 | 168 | 1 | 84,629 |
| 4 | + | 40 | 0 | 1 | 88,356 |
| 5 | + | 40 | 10 | 1 | 85,949 |
| 6 | + | 40 | 168 | 1 | 83,085 |
| 7 | + | 20 | 0 | 2 | 33,949 |
| 8 | + | 20 | 10 | 2 | 94,977 |
| 9 | + | 20 | 168 | 2 | 93,648 |
| 10 | + | 40 | 0 | 2 | 33,949 |
| 11 | + | 40 | 10 | 2 | 90,261 |
| 12 | + | 40 | 168 | 2 | 75,818 |
| 13 | + | 20 | 0 | 3 | 166,679 |
| 14 | + | 20 | 10 | 3 | 163,693 |
| 15 | + | 20 | 168 | 3 | 174,793 |
| 16 | + | 40 | 0 | 3 | 166,679 |
| 17 | + | 40 | 10 | 3 | 101,247 |
| 18 | + | 40 | 168 | 3 | 173,461 |
| 19 | - | 20 | 0 | 1 | 83,372 |
| 20 | - | 20 | 10 | 1 | 61,723 |
| 21 | - | 20 | 168 | 1 | 46,404 |
| 22 | - | 40 | 0 | 1 | 83,372 |
| 23 | - | 40 | 10 | 1 | 27,798 |
| 24 | - | 40 | 168 | 1 | 10,678 |
| 25 | - | 20 | 0 | 2 | 99,792 |
| 26 | - | 20 | 10 | 2 | 95,167 |
| 27 | - | 20 | 168 | 2 | 63,769 |
| 28 | - | 40 | 0 | 2 | 99,792 |

Appendix Table 1 (continued). The results of quantitative PCR for tank experiments (Validation 1).

| ID | ATL | Preserved temperature  [℃] | Preserved time  [h] | # Tank | eDNA concentration  [copies/reaction] |
| --- | --- | --- | --- | --- | --- |
| 29 | - | 40 | 10 | 2 | 70,049 |
| 30 | - | 40 | 168 | 2 | 15,931 |
| 31 | - | 20 | 0 | 3 | 190,833 |
| 32 | - | 20 | 10 | 3 | 175,976 |
| 33 | - | 20 | 168 | 3 | 90,315 |
| 34 | - | 40 | 0 | 3 | 190,833 |
| 35 | - | 40 | 10 | 3 | 105,107 |
| 36 | - | 40 | 168 | 3 | 44,049 |

Appendix Table 2. The results of quantitative PCR for river experiments (Validation 2).

| ID | ATL | Preserved temperature  [℃] | Preserved time  [h] | eDNA concentration  [copies/reaction] |
| --- | --- | --- | --- | --- |
| 1 | + | 20 | 0 | 1,118 |
| 2 | + | 20 | 0 | 754 |
| 3 | + | 20 | 0 | 777 |
| 4 | + | 20 | 10 | 788 |
| 5 | + | 20 | 10 | 972 |
| 6 | + | 20 | 10 | 836 |
| 7 | + | 20 | 168 | 936 |
| 8 | + | 20 | 168 | 899 |
| 9 | + | 20 | 168 | 938 |
| 10 | + | 40 | 0 | 1,118 |
| 11 | + | 40 | 0 | 754 |
| 12 | + | 40 | 0 | 777 |
| 13 | + | 40 | 10 | 858 |
| 14 | + | 40 | 10 | 836 |
| 15 | + | 40 | 10 | 913 |
| 16 | + | 40 | 168 | 1,171 |
| 17 | + | 40 | 168 | 1,225 |
| 18 | + | 40 | 168 | 862 |
| 19 | - | 20 | 0 | 1,306 |
| 20 | - | 20 | 0 | 724 |
| 21 | - | 20 | 0 | 734 |
| 22 | - | 20 | 10 | 776 |
| 23 | - | 20 | 10 | 770 |
| 24 | - | 20 | 10 | 697 |
| 25 | - | 20 | 168 | 512 |
| 26 | - | 20 | 168 | 742 |
| 27 | - | 20 | 168 | 689 |
| 28 | - | 40 | 0 | 1,306 |
| 29 | - | 40 | 0 | 724 |
| 30 | - | 40 | 0 | 734 |

Appendix Table 2 (continued). The results of quantitative PCR for river experiments (Validation 2).

| ID | ATL | Preserved temperature  [℃] | Preserved time  [h] | eDNA concentration  [copies/reaction] |
| --- | --- | --- | --- | --- |
| 31 | - | 40 | 10 | 757 |
| 32 | - | 40 | 10 | 696 |
| 33 | - | 40 | 10 | 908 |
| 34 | - | 40 | 168 | 263 |
| 35 | - | 40 | 168 | 438 |
| 36 | - | 40 | 168 | 501 |

Appendix Table 3. Species detected by eDNA metabarcoding for river experiments (Validation 2).

| No. | Latin name |
| --- | --- |
| 1 | *Rhinogobius* sp. |
| 2 | *Nipponocypris temminckii* |
| 3 | *Misgurnus* sp. |
| 4 | *Rhynchocypris jouyi* |
| 5 | *Tridentiger* sp. |
| 6 | *Anguilla japonica* |
| 7 | *Plecoglossus altivelis* |
| 8 | *Gymnogobius* sp. |
| 9 | *Oncorhynchus* sp. |
| 10 | *Acanthogobius flavimanus* |
| 11 | *Luciogobius* sp. |
| 12 | *Gnathopogon elongatus* |
| 13 | *Redigobius* sp. |
| 14 | *Cyprinus carpio* |
| 15 | *Opsariichthys platypus* |
| 16 | *Gadus* sp.* |
| 17 | *Hemiculter leucisculus* |
| 18 | *Pseudaspius hakonensis* |
| 19 | *Hemibarbus* sp. |
| 20 | *Micropterus* sp. |
| 21 | *Carassius* sp. |
| 22 | *Favonigobius gymnauchen** |
| 23 | *Saurida* sp. * |
| 24 | *Mugil cephalus* |

An asterisk (*) indicates marine fish

Appendix Table 4. Number of fish species detected by metabarcoding from each sample in the field survey at the Sumiyoshi River (Validation 2)

| ID | ATL | Preserved temperature  [℃] | Preserved time  [h] | Number of detected species |
| --- | --- | --- | --- | --- |
| 1 | + | 20 | 0 | 12 |
| 2 | + | 20 | 0 | 10 |
| 3 | + | 20 | 0 | 10 |
| 4 | + | 20 | 10 | 12 |
| 5 | + | 20 | 10 | 12 |
| 6 | + | 20 | 10 | 10 |
| 7 | + | 20 | 168 | 13 |
| 8 | + | 20 | 168 | 12 |
| 9 | + | 20 | 168 | 11 |
| 10 | + | 40 | 0 | 12 |
| 11 | + | 40 | 0 | 10 |
| 12 | + | 40 | 0 | 10 |
| 13 | + | 40 | 10 | 11 |
| 14 | + | 40 | 10 | 11 |
| 15 | + | 40 | 10 | 11 |
| 16 | + | 40 | 168 | 12 |
| 17 | + | 40 | 168 | 10 |
| 18 | + | 40 | 168 | 12 |
| 19 | - | 20 | 0 | 10 |
| 20 | - | 20 | 0 | 12 |
| 21 | - | 20 | 0 | 13 |
| 22 | - | 20 | 10 | 10 |
| 23 | - | 20 | 10 | 12 |
| 24 | - | 20 | 10 | 12 |
| 25 | - | 20 | 168 | 12 |
| 26 | - | 20 | 168 | 11 |
| 27 | - | 20 | 168 | 11 |
| 28 | - | 40 | 0 | 10 |
| 29 | - | 40 | 0 | 12 |
| 30 | - | 40 | 0 | 13 |

Appendix Table 4 (continued). Number of fish species detected by metabarcoding from each sample in the field survey at the Sumiyoshi River (Validation 2)

| ID | ATL | Preserved temperature  [℃] | Preserved time  [h] | Number of detected species |
| --- | --- | --- | --- | --- |
| 31 | - | 40 | 10 | 12 |
| 32 | - | 40 | 10 | 11 |
| 33 | - | 40 | 10 | 12 |
| 34 | - | 40 | 168 | 11 |
| 35 | - | 40 | 168 | 14 |
| 36 | - | 40 | 168 | 11 |

The sample IDs correspond to Appendix Table 2

| Species / Sample ID | 1 | 2 | 3 | 4 | 5 | 6 | 7 | 8 | 9 | 10 | 11 | 12 | 13 | 14 | 15 |
| --- | --- | --- | --- | --- | --- | --- | --- | --- | --- | --- | --- | --- | --- | --- | --- |
| *Rhinogobius* sp. | 32898 | 20320 | 16422 | 22512 | 22216 | 23555 | 20460 | 24655 | 23821 | 19941 | 13630 | 24918 | 22705 | 27714 | 22798 |
| *Nipponocypris temminckii* | 8389 | 5250 | 5369 | 5527 | 6030 | 7797 | 5517 | 6041 | 6096 | 4476 | 3424 | 8187 | 6495 | 7749 | 6463 |
| *Misgurnus* sp. | 7275 | 5725 | 4292 | 5063 | 5654 | 5810 | 4879 | 5867 | 6038 | 3156 | 3344 | 6261 | 6230 | 5458 | 5100 |
| *Rhynchocypris jouyi* | 9797 | 6545 | 3860 | 5894 | 5283 | 5076 | 3686 | 5521 | 5660 | 4186 | 2578 | 4322 | 4703 | 6109 | 5468 |
| *Tridentiger* sp. | 3528 | 407 | 803 | 505 | 1103 | 650 | 491 | 650 | 945 | 316 | 487 | 945 | 1089 | 1040 | 1598 |
| *Anguilla japonica* | 1541 | 1425 | 900 | 845 | 1195 | 1424 | 936 | 1935 | 1122 | 835 | 437 | 2001 | 1702 | 1484 | 1917 |
| *Plecoglossus altivelis* | 1067 | 603 | 598 | 378 | 676 | 668 | 497 | 563 | 637 | 346 | 314 | 998 | 641 | 925 | 950 |
| *Gymnogobius* sp. | 624 | 814 | 400 | 341 | 713 | 106 | 919 | 421 | 792 | 742 | 41 | 231 | 477 | 500 | 458 |
| *Oncorhynchus* sp. | 325 | 0 | 0 | 116 | 54 | 0 | 78 | 0 | 0 | 0 | 0 | 0 | 0 | 63 | 0 |
| *Acanthogobius flavimanus* | 116 | 0 | 0 | 0 | 50 | 0 | 64 | 0 | 87 | 0 | 29 | 0 | 194 | 0 | 0 |
| *Luciogobius* sp. | 98 | 49 | 193 | 36 | 49 | 298 | 123 | 138 | 193 | 45 | 29 | 76 | 160 | 184 | 224 |
| *Gnathopogon elongatus* | 57 | 0 | 0 | 0 | 0 | 0 | 0 | 28 | 0 | 0 | 0 | 0 | 0 | 0 | 0 |
| *Redigobius* sp. | 0 | 25 | 55 | 49 | 8 | 0 | 24 | 46 | 15 | 18 | 55 | 0 | 133 | 0 | 0 |
| *Cyprinus carpio* | 0 | 0 | 0 | 33 | 0 | 112 | 0 | 0 | 0 | 39 | 0 | 41 | 0 | 0 | 0 |
| *Opsariichthys platypus* | 0 | 0 | 0 | 0 | 0 | 0 | 27 | 0 | 0 | 0 | 0 | 0 | 26 | 0 | 0 |

Appendix Table 5. Read counts of each fish species detected from samples in the Sumiyoshi River

Appendix Table 5 (continued). Read counts of each fish species detected from samples in the Sumiyoshi River

| Species / Sample ID | 1 | 2 | 3 | 4 | 5 | 6 | 7 | 8 | 9 | 10 | 11 | 12 | 13 | 14 | 15 |
| --- | --- | --- | --- | --- | --- | --- | --- | --- | --- | --- | --- | --- | --- | --- | --- |
| *Gadus* sp. | 0 | 0 | 0 | 0 | 0 | 0 | 0 | 40 | 0 | 0 | 0 | 0 | 0 | 0 | 0 |
| *Hemiculter leucisculus* | 0 | 0 | 0 | 0 | 0 | 0 | 0 | 0 | 0 | 0 | 0 | 0 | 0 | 0 | 0 |
| *Pseudaspius hakonensis* | 0 | 0 | 0 | 0 | 0 | 0 | 0 | 0 | 0 | 0 | 0 | 0 | 0 | 0 | 0 |
| *Hemibarbus* sp. | 0 | 0 | 0 | 0 | 0 | 0 | 0 | 0 | 0 | 0 | 0 | 0 | 0 | 0 | 0 |
| *Micropterus* sp. | 0 | 0 | 0 | 0 | 0 | 0 | 0 | 0 | 0 | 0 | 0 | 0 | 0 | 0 | 42 |
| *Carassius* sp. | 0 | 0 | 0 | 0 | 0 | 0 | 0 | 0 | 0 | 0 | 0 | 0 | 0 | 0 | 0 |
| *Favonigobius gymnauchen* | 0 | 0 | 0 | 0 | 0 | 0 | 0 | 0 | 0 | 0 | 0 | 26 | 0 | 0 | 0 |
| *Saurida* sp. | 0 | 0 | 0 | 0 | 0 | 0 | 0 | 0 | 0 | 0 | 0 | 0 | 0 | 0 | 142 |
| *Mugil cephalus* | 0 | 0 | 0 | 0 | 0 | 0 | 0 | 0 | 0 | 0 | 0 | 0 | 0 | 0 | 36 |

Appendix Table 5 (continued). Read counts of each fish species detected from samples in the Sumiyoshi River

| Species / Sample ID | 16 | 17 | 18 | 19 | 20 | 21 | 22 | 23 | 24 | 25 | 26 | 27 | 28 | 29 | 30 |
| --- | --- | --- | --- | --- | --- | --- | --- | --- | --- | --- | --- | --- | --- | --- | --- |
| *Rhinogobius* sp. | 20090 | 17212 | 16789 | 19549 | 19678 | 18720 | 21112 | 13388 | 13681 | 12432 | 14387 | 16654 | 17711 | 16321 | 27367 |
| *Nipponocypris temminckii* | 4938 | 4722 | 4593 | 4460 | 5255 | 5196 | 5219 | 4578 | 3560 | 3483 | 3354 | 4055 | 6905 | 3597 | 6797 |
| *Misgurnus* sp. | 3686 | 3801 | 3532 | 5094 | 4147 | 3596 | 5504 | 13090 | 3425 | 3464 | 3434 | 5004 | 3818 | 4879 | 6332 |
| *Rhynchocypris jouyi* | 4273 | 3965 | 3096 | 3862 | 4426 | 4619 | 896 | 1750 | 3131 | 2079 | 2760 | 3015 | 2267 | 2934 | 3695 |
| *Tridentiger* sp. | 316 | 561 | 538 | 731 | 980 | 546 | 1709 | 611 | 477 | 805 | 680 | 778 | 0 | 969 | 1921 |
| *Anguilla japonica* | 1662 | 719 | 686 | 239 | 1009 | 1208 | 1821 | 1253 | 631 | 776 | 628 | 1193 | 524 | 899 | 942 |
| *Plecoglossus altivelis* | 1046 | 423 | 320 | 455 | 827 | 1002 | 976 | 834 | 370 | 604 | 359 | 546 | 524 | 679 | 906 |
| *Gymnogobius* sp. | 572 | 413 | 328 | 221 | 289 | 82 | 1179 | 350 | 462 | 223 | 796 | 334 | 801 | 1311 | 882 |
| *Oncorhynchus* sp. | 0 | 0 | 0 | 0 | 127 | 0 | 0 | 0 | 0 | 0 | 0 | 101 | 0 | 70 | 0 |
| *Acanthogobius flavimanus* | 0 | 115 | 73 | 0 | 265 | 54 | 137 | 28 | 45 | 142 | 102 | 79 | 675 | 0 | 186 |
| *Luciogobius* sp. | 37 | 26 | 78 | 45 | 251 | 205 | 117 | 200 | 109 | 123 | 56 | 74 | 0 | 162 | 322 |
| *Gnathopogon elongatus* | 0 | 0 | 0 | 0 | 0 | 0 | 0 | 0 | 0 | 69 | 0 | 0 | 0 | 120 | 0 |
| *Redigobius* sp. | 71 | 57 | 130 | 118 | 42 | 76 | 279 | 56 | 7 | 56 | 124 | 29 | 96 | 117 | 51 |
| *Cyprinus carpio* | 0 | 66 | 0 | 0 | 0 | 95 | 0 | 0 | 0 | 0 | 0 | 0 | 0 | 0 | 0 |
| *Opsariichthys platypus* | 0 | 0 | 80 | 0 | 0 | 0 | 0 | 0 | 0 | 0 | 0 | 0 | 382 | 0 | 0 |

Appendix Table 5 (continued). Read counts of each fish species detected from samples in the Sumiyoshi River

| Species / Sample ID | 16 | 17 | 18 | 19 | 20 | 21 | 22 | 23 | 24 | 25 | 26 | 27 | 28 | 29 | 30 |
| --- | --- | --- | --- | --- | --- | --- | --- | --- | --- | --- | --- | --- | --- | --- | --- |
| *Gadus* sp. | 0 | 0 | 0 | 0 | 0 | 0 | 0 | 0 | 0 | 0 | 0 | 0 | 0 | 0 | 0 |
| *Hemiculter leucisculus* | 0 | 0 | 0 | 0 | 0 | 0 | 0 | 0 | 0 | 0 | 0 | 0 | 197 | 0 | 0 |
| *Pseudaspius hakonensis* | 0 | 0 | 0 | 0 | 0 | 0 | 0 | 0 | 0 | 0 | 0 | 0 | 0 | 142 | 0 |
| *Hemibarbus* sp. | 0 | 0 | 0 | 0 | 0 | 0 | 0 | 0 | 0 | 0 | 0 | 0 | 0 | 104 | 0 |
| *Micropterus* sp. | 0 | 0 | 37 | 0 | 0 | 0 | 0 | 0 | 0 | 0 | 0 | 0 | 0 | 0 | 0 |
| *Carassius* sp. | 0 | 0 | 0 | 0 | 0 | 0 | 84 | 0 | 0 | 0 | 0 | 0 | 0 | 0 | 0 |
| *Favonigobius gymnauchen* | 0 | 0 | 0 | 0 | 0 | 0 | 0 | 0 | 0 | 0 | 0 | 0 | 0 | 0 | 0 |
| *Saurida* sp*.* | 0 | 0 | 0 | 0 | 0 | 0 | 0 | 0 | 0 | 0 | 0 | 0 | 0 | 0 | 0 |
| *Mugil cephalus* | 0 | 0 | 0 | 0 | 0 | 0 | 0 | 0 | 0 | 0 | 0 | 0 | 0 | 0 | 0 |

The samples IDs correspond to Appendix Table 2

Appendix Table 6. Read counts of each fish species detected from the blank samples

| Species | Field blank | NTC1 | NTC2 |
| --- | --- | --- | --- |
| *Rhinogobius* sp. | 57 | 507 | 654 |
| *Nipponocypris temminckii* | 18 | 123 | 186 |
| *Misgurnus* sp. | 12 | 94 | 139 |
| *Rhynchocypris jouyi* | 12 | 85 | 125 |
| *Tridentiger* sp. | 0 | 20 | 42 |
| *Anguilla japonica* | 0 | 33 | 25 |
| *Plecoglossus altivelis* | 0 | 21 | 24 |
| *Gymnogobius* sp. | 0 | 12 | 11 |
| *Oncorhynchus* sp. | 0 | 0 | 0 |
| *Acanthogobius flavimanus* | 0 | 0 | 0 |
| *Luciogobius* sp. | 0 | 0 | 0 |
| *Gnathopogon elongatus* | 0 | 0 | 0 |
| *Redigobius* sp. | 0 | 0 | 0 |
| *Cyprinus carpio* | 0 | 0 | 0 |
| *Opsariichthys platypus* | 0 | 0 | 0 |
| *Gadus* sp. | 0 | 0 | 0 |
| *Hemiculter leucisculus* | 0 | 0 | 0 |
| *Pseudaspius hakonensis* | 0 | 0 | 0 |
| *Hemibarbus* sp. | 0 | 0 | 0 |
| *Micropterus* sp. | 0 | 0 | 0 |
| *Carassius* sp. | 0 | 0 | 0 |
| *Favonigobius gymnauchen* | 0 | 0 | 0 |
| *Saurida* sp. | 0 | 0 | 0 |
| *Mugil cephalus* | 0 | 0 | 0 |
